# Introducing a low-risk breast screening pathway into the NHS Breast Screening Programme: Views from healthcare professionals who are delivering risk-stratified screening

**DOI:** 10.1177/17455065211009746

**Published:** 2021-04-20

**Authors:** Victoria G Woof, Lorna McWilliams, Louise S Donnelly, Anthony Howell, D Gareth Evans, Anthony J Maxwell, David P French

**Affiliations:** 1 Manchester Centre for Health Psychology, Division of Psychology & Mental Health, School of Health Sciences, Faculty of Biology, Medicine and Health, University of Manchester, MAHSC, Manchester, UK; 2 Nightingale and Prevent Breast Cancer Research Unit, Manchester University NHS Foundation Trust, Manchester, UK; 3 NIHR Greater Manchester Patient Safety Translational Research Centre, Centre for Mental Health and Safety, School of Health Sciences, Faculty of Biology, Medicine and Health, University of Manchester, MAHSC, Manchester, UK; 4 Department of Genomic Medicine, Division of Evolution and Genomic Science, University of Manchester, MAHSC, Manchester University NHS Foundation Trust, Manchester, UK; 5 Division of Informatics, Imaging & Data Sciences, School of Health Sciences, Faculty of Biology, Medicine and Health, University of Manchester, Manchester, UK

**Keywords:** healthcare professionals, low-risk, risk estimation, risk-stratified breast screening, screening frequency

## Abstract

**Objectives:**

Proposals to stratify breast screening by breast cancer risk aim to produce a better balance of benefits to harms. Notably, risk estimation calculated from common risk factors and a polygenic risk score would enable high-risk women to benefit from more frequent screening or preventive medication. This service would also identify low-risk women who experience fewer benefits from attending, as lower grade and in situ cancers may be treated unnecessarily. It may therefore be appropriate for low-risk women to attend screening less. This study aimed to elicit views regarding implementing less frequent screening for low-risk women from healthcare professionals who implement risk-stratified screening.

**Methods:**

Healthcare professionals involved in the delivery of risk-stratified breast screening were invited to participate in a focus group within the screening setting in which they work or have a telephone interview. Primary care staff were also invited to provide their perspective. Three focus groups and two telephone interviews were conducted with 28 healthcare professionals. To identify patterns across the sample, data were analysed as a single dataset using reflexive thematic analysis.

**Results:**

Analysis yielded three themes: *Reservations concerning the introduction of less frequent screening*, highlighting healthcare professionals’ unease and concerns towards implementing less frequent screening; *Considerations for the management of public knowledge*, providing views on media impact on public opinion and the potential for a low-risk pathway to cause confusion and raise suspicion regarding implementation motives; and *Deliberating service implications and reconfiguration management*, where the practicalities of implementation are discussed.

**Conclusions:**

Healthcare professionals broadly supported less frequent screening but had concerns about implementation. It will be essential to address concerns regarding risk estimate accuracy, healthcare professional confidence, service infrastructure and public communication prior to introducing less frequent screening for low-risk women.

## Introduction

Population-based breast cancer screening utilizes a ‘one-size-fits-all’ model, where most women within particular age ranges are screened with the same interval.^1^ Screening aims to identify cancers at an early stage to reduce the mortality rate and minimize treatment. It is argued by some that the benefits of screening outweigh the harms, such as false-positive screens and over-diagnosis leading to overtreatment.^2^ In the United Kingdom, it has been estimated that for every life saved from breast screening, three women are overdiagnosed.^3^ For some, this balance of benefits to harms is too modest.^4^

One way to improve the balance of benefits to harms is to introduce a more personalized breast screening service, whereby the screening interval is based on the individual’s risk of developing breast cancer.^5^ It is possible to estimate the probability of breast cancer by calculating a polygenic risk score from the combined effect of single nucleotide polymorphisms (SNPs) related to the disease.^6,7^ This genetic information combined with common risk factors, such as family history, reproductive factors and mammographic density allows for an accurate breast cancer risk estimate. A key benefit of risk estimation and a risk-stratified breast screening service is the ability to identify women at higher risk, affording them the opportunity to benefit from more frequent screening and preventive medications.^8^ Therefore, there are efforts globally to introduce risk stratification into national breast screening programmes.^9–12^

For women at low risk, the benefits of breast screening are less clear, as screening harms may outweigh benefits.^13,14^ To minimize these harms, it has been proposed that the frequency at which low-risk women attend for screening could be reduced, with some proposing withdrawing breast screening entirely for this risk population.^13–16^ To date, a small body of evidence suggests it could be safe to alter the frequency at which low-risk women attend for breast screening.^5,16,17^ Where women have been approached with the idea to alter screening for those at low risk, a complete withdrawal of screening has been opposed.^18–20^ However, perceptions towards less frequent screening have been more favourable, providing the change is appropriately evidenced and clearly communicated.^18,21^

Implementing a low-risk screening pathway also needs to be acceptable to healthcare professionals (HCPs) working in breast screening and primary care. Where views have been sought about risk stratification, HCPs have expressed concerns regarding implementation, including HCP responsibilities for communicating risk and the proposed benefits to women, especially for those who are ‘near’ population risk.^22,23^ It has yet to be established what HCPs working across the breast screening service and primary care think of introducing a risk-stratified breast screening programme which involves screening low-risk women less often. It is therefore important to investigate the views of HCPs towards such a change, as their views will influence the success of adaptations to healthcare services.^24^ Such investigation could thereby allow any implementation of a low-risk screening pathway to take account of these views in either pathway design, or through appropriate training. Consideration of the views of HCPs may be particularly warranted in the present context as professionals working in screening programmes generally want to increase screening uptake to allow more individuals to benefit from disease being detected earlier, albeit with a recognition of the harms of screening. By contrast, a low-risk screening pathway could be considered at odds with this view, as it aims to decrease the amount of screening, with a strong focus on screening harms.

The present study is the first, to our knowledge, to explore HCP views regarding less frequent breast screening for women at low risk. HCPs currently involved in implementing risk-stratified breast screening in the NHS Breast Screening Programme (NHSBSP) as part of a feasibility study (BC-Predict)^9^ were asked to provide their opinions on the acceptability and feasibility of including a low-risk screening pathway into the UK National Health Service Breast Screening Programme (NHSBSP). Primary care HCPs who were local to the screening sites were also invited to share their views.

## Methods

### Design

A cross-sectional design was adopted employing focus groups (FGs) and one-to-one semi-structured telephone interviews.

### Participants

HCPs involved in organizing and implementing the NHSBSP and local primary care clinicians were purposively sampled across three breast screening sites in North-West England where risk-stratified breast screening is currently being implemented as part of a feasibility study (BC-Predict).^9^ In this study, participants are provided with a 10-year risk estimate.^9^ A proportion of the HCPs recruited to this study also took part in a focus group a year earlier to examine their views on risk-stratified screening in general as part of BC-Predict.^9,25^

Twenty-eight HCPs took part: three male and 25 female. Table 1 details the professional roles represented.

**Table 1.** Focus group/interview participant occupations.

Occupation	Participants (n = 28)
Radiographer breast imaging manager	1
Superintendent radiographer/programme manager	1
Breast screening office manager	1
Breast care nurse	1
Admin and data clerk	1
General practitioner^a^	3
Trainee mammographer	1
Radiographer or mammographer (describes the same role)	9
Cancer screening improvement lead	2
Advanced practitioner radiography/mammography (describes the same role)	5
Consultant radiologist	3

a One General Practitioner also has a commissioning role.

### Procedure

Participants were recruited via Breast Screening Office Managers who distributed information (i.e. the participant information sheet) to their staff physically and via email. A study invitation and study information were sent via post to General Practitioner (GP) practices local to the breast screening study sites to recruit participants from primary care. The study team also approached primary care professionals who were known to them via email. Interested participants were asked to contact the study team if they wanted to take part in a focus group or interview to discuss the feasibility of increasing the screening interval for women at low risk of breast cancer.

FGs were chosen as the preferred method of data collection as the environment encourages lively debate and exploration of contradictions among members to facilitate a thorough appraisal of the discussion topic.^26^ Three FGs were conducted (n = 11, n = 7 and n = 8 for each FG), one at each screening site, each lasting 1–2 hours. Two female researchers (VGW, LSD or LM) with postgraduate qualitative health services research experience were present at each focus group. One researcher acted as moderator and the other to take notes and manage the audio-recorder. All participants provided informed written consent prior to focus group or interviews commencing. A semi-structured topic guide was used flexibly throughout the FGs and interviews to keep the discussion focused and provoke debate. This guide was developed by members of the research team (VGW, LM & DPF) through multiple discussions, creating various iterations before finalizing questions. Questions related to how a low-risk pathway could be implemented and organized and what effect this pathway could have on HCPs, women in the general population and overall public opinion. One-to-one interviews were held (VGW) with two GPs who were unable to attend a focus group using the same topic guide. Data were transcribed by an external transcription company.

### Analysis

Data were analysed in NVivo11 using reflexive thematic analysis, a method which acknowledges the role of researcher subjectivity in the production of the final thematic structure.^27,28^ A realist perspective to the analysis was adopted, in that the researchers accepted participant’s experiences and views as reflecting their truth and reality. Primary data analyses were conducted by VGW and LM. Transcripts were read and audio files listened to multiple times to allow the researchers to become familiar with the data. Interview data were analysed with the FG data as a single dataset. During this process, notes of initial ideas and points of interest were taken to aid familiarization of data and reflect on in later analysis stages. Following familiarization, coding began at an inductive-manifest level where there was no pre-existing coding framework. This form of coding facilitates the production of a rich and data-driven analysis, mitigating the impact of existing theory and literature. An initial FG transcript was independently coded line by line by two authors (VGW, LM) to discuss early views about the data rather than to check accuracy or reliability. Coding was then approached iteratively, with initial points of interest identified within the data and used to label the codes. The codes were used to identify patterns across the dataset. As coding became more refined, initial themes were developed with codes organized into subthemes that contribute to the overall understanding of each theme.^29^ Codes and themes were continually and collaboratively refined by VGW, LM and DPF to develop a final thematic structure deemed representative of the participants’ expressed views and experiences about the acceptability and feasibility of risk-stratified screening.

## Results

The reflexive thematic analysis produced three themes: (1) Reservations concerning the introduction of less frequent screening; (2) Considerations for the management of public knowledge; and (3) Deliberating service implications and reconfiguration management. Quotes presented are anonymised by the use of professional role (found in Table 1).

### Theme 1 – Reservations concerning the introduction of less frequent screening

Overall, HCPs believed that reduced screening for low-risk women was, in principle, logical but were cautious. HCPs were sceptical that risk estimates were likely to be accurate and stable enough to be confidently used to introduce a low-risk screening pathway. HCPs described feeling uneasy about advocating less frequent screening because of the possibility that more interval cancers would result. They also questioned how women would perceive being at low risk and were concerned that some would assume themselves to be at no risk.

#### Subtheme 1.1. Low-risk screening is logical in theory

Overall, HCPs believed that risk-stratified screening is the next logical step for breast screening, and that reduced screening for low-risk women was logical *in theory*. They suggested that the NHSBSP should question whether a ‘one-size-fits-all’ model remains appropriate if evidence suggests that screening harms can be reduced for low-risk women by screening them less frequently. They explained that the breast screening service receives criticism for the harms it can cause and that a risk-stratified service would go some way to address this perception:

I think we get criticised all the time for overtreatment and over diagnosis and we should be seen to be trying to personalise it a bit more, but we shouldn’t overthink it and overcomplicate it in the process. (Consultant Radiologist: FG3)

Therefore, HCPs agreed that risk-stratified screening would provide women with a more personal service, which has a better balance of benefits to harms for all risk groups:

I think the idea is really good personally because I’d like to think that the women that have really, really low risk that they are . . . there is a long period of time between screening [. . .] I think that would be really good, and then we concentrate then on getting those ones that are high risk in. I mean, it makes sense, doesn’t it? (Superintendent Radiographer: FG2)

Yeah, of course it does. (Radiographer: FG2)

Nevertheless, despite positive reactions towards risk stratification, reduced screening was still discussed cautiously, with many questions raised about the difficulties of implementation.

#### Subtheme 1.2. Questioning the reliability of risk

HCPs stressed that confidence in risk estimate accuracy will be essential if low-risk women are to attend screening less frequently. They raised questions regarding the stability of a 10-year risk estimate with concerns about how quickly it could change and subsequently affect the frequency at which women should be screened. They cited the increased risk of breast cancer with age, changes in mammographic density, the inclusion or exclusion of a polygenic risk score and the development of breast cancer in relatives as factors which could change a risk estimate:

How do we get around the fact that everybody’s risk increases as they get older? So if you give somebody their risk at 47 when they first come, when is that reviewed? Because we know that the majority of cancers occur in the older cohort of ladies, so your risk just increases as you get older anyway. So do you then at your six or your 12 year mammogram have another density reading and your risk is . . .? (Breast Screening Office Manager: FG1)

Issues were also raised about the accuracy of women’s self-reported information used to calculate risk. With this in mind, HCPs were apprehensive about implementing less frequent screening if categorisation for some is based on inaccurate information:

I’m just thinking about those who might think, right, okay, I’ve got a low-risk, but what if circumstances change? And sometimes they might have breast cancer in the family and they might not know, because a lot of women don’t tell. (Cancer Screening Improvement Lead: FG3)

If risk can change markedly HCPs explained that a risk reassessment service is needed and is especially important for low-risk women, where screening may not be as frequent so cancers could remain undetected for longer. However, HCPs were unclear about frequency of reassessment.

#### Subtheme 1.3. Unease towards providing screening frequency advice

HCPs debated whether women at low risk of breast cancer should be able to choose their screening frequency. They explained that the NHSBSP provides reassurance for many women and reducing screening automatically would take this reassurance away. Therefore, some advocated that reduced screening should be a choice but others explained that many women would be overwhelmed by the information needed to make an informed decision and would instead seek support from HCPs and the service. They acknowledged the trust women have in HCPs to make decisions about their health on their behalf:

…I think sometimes people just want to know what we think, that’s a very, very powerful part of the equation. If you’re saying to people, well, I think it’s a good idea because of this, they trust that, they accept that because they trust us. (GP: TI 1)

With this in mind, HCPs expressed unease about advocating less frequent screening for those at low risk. In particular, they would feel uncomfortable facilitating decisions due to the occurrence of interval cancers:

I wouldn’t feel comfortable in telling somebody to have a longer gap in the screening if I wasn’t 100 per cent that [. . .] I personally wouldn’t be like, well, yeah, just leave it five years because I’d be really conscious of them developing a cancer in between. (Mammographer: FG3)

On a personal level, HCPs expressed concern that low-risk women who develop breast cancer during a longer interval would blame the HCP who advised them: ‘I don’t want that to come back on us, but you don’t want to feel like we’ve done this’ (Radiographer: FG2). Alternatively some explained that providing women with choice of screening frequency would defeat the purpose of a risk-stratified service. If less frequent screening for low-risk women were mandatory, HCPs would not be required to facilitate women’s decisions, potentially reducing unease about providing advice.

#### Subtheme 1.4. Low risk is not ‘no risk’

HCPs had qualms about how women would interpret a low-risk estimate. They emphasized that some women could misattribute being at low-risk as having ‘no risk’, especially if advised to attend screening less often:

So people find it difficult to understand risk. So if you say, for example, to a lady, oh, you don’t have to come for five years, but they might think, oh, I won’t get breast cancer because I’m such a low risk so I won’t get it. (Advanced Practitioner – Mammography: FG2)

HCPs expressed that this misperception could cause low-risk women to be less breast aware and fail to address symptoms: ‘My worry would be if you tell someone they’ve got a low risk of breast cancer they’re going to ignore symptoms . . .’ (Consultant Radiologist: FG1). Doubts were also raised about whether low-risk women would attend subsequent screening appointments where the NHSBSP could see reduced uptake should these women feel the service is no longer applicable:

So if you went for the initial screening for breast and you were classed as low risk, then you might think, I won’t bother again then . . . (Cancer Screening Improvement Lead: FG3)

As cancers can still develop in low-risk women, HCPs stressed the importance of providing clear information to women detailing that low risk does not indicate an immunity to developing breast cancer and breast awareness remains essential.

### Theme 2 – Considerations for the management of public knowledge

HCP’s unease towards less frequent screening for low-risk women also influenced discussions about media representations and public opinions towards a low-risk pathway. They explored the media’s power in shaping public opinion, both positively and negatively, and its potential for educating the public. HCPs highlighted that the public may view screening low-risk women as a cost-saving strategy and suggested that inconsistent messaging from the breast screening services and community networks could negatively impact screening behaviour and women’s understanding of changes.

#### Subtheme 2.1. Navigating media output

HCPs explained that media output can ‘make or break’ public opinion, especially when communication focuses on changes to NHS services. When considering the implementation of a low-risk screening pathway, some HCPs felt that the media could focus on the negatives, such as interval cancers and potential deaths from breast cancer. This was especially an issue for implementing the pilot phase where not all screening units are involved and underpinning evidence is still being gathered:

If it’s rolled out across the NHSBSP because there’s hard evidence this is what everyone’s going to do it’s not a problem. But all of a sudden you’ve got newspapers saying reduced screening in London, three ladies die because of it, whereas if you go to Birmingham they wouldn’t do. I think that’s something we have to be very mindful of, particularly if you’re looking at pilot sites doing it. (Consultant Radiologist: FG1)

On the other hand, others explained that less frequent screening for low-risk women could be presented positively in the media. HCPs identified that the NHSBSP is often criticized for its harms, and so measures to reduce these by safely screening low-risk women less often could address this negative perception:

Because it’s always in the press about the harm from breast screening. It’s always in the press, are we overreacting, over diagnosing? So if you could go out to the press and say, well, to reduce that by this much, we are going to stop screening this group of women every three years and do them every six years, and therefore we’ll have 50 per cent reduction in the unnecessary whatever, and it’s just whatever spin you put on it. (Consultant Radiologist: FG3)

HCPs explained that for the public to receive balanced reporting about the benefits and harms of less frequent screening, the media will need clear and consistent communication from the service about proposed changes and their effect on women.

#### Subtheme 2.2. Navigating public scrutiny

HCPs identified that the public may not view an extended screening interval for low-risk women favourably. There was unease that the public would view this as a cost-saving measure rather than for the benefit of the population: ‘I think sometimes patients worry that we’re doing things purely on cost rather than on quality’ (GP: TI2). HCPs suggested that public concern surrounding ‘cost-cutting’ would need to be explicitly acknowledged and explained. For example, it would be reasonable for the public to know that changes are partially due to ‘reallocation of resources’ to support those at high risk, but safety and a reduction in screening harms ought to be communicated as the primary motivation for screening low-risk women less. However, HCPs acknowledged that the public as a whole are not cognisant of screening harms and could find it difficult to appreciate why less frequent screening is being recommended:

I mean it’s quite a subtle message, isn’t it? For years and years we’ve been telling ladies you must go and have your screenings, and I think screening in the public mind is very much wrapped around screening is good always. I think it’s very hard to discuss subtleties of potential screening harms with people. (GP: TI1)

To mitigate public scrutiny and facilitate understanding, HCPs explained that the personal benefit of risk-stratified breast screening will need to be widely emphasized. HCPs advocated working with media outlets and charities to create public education initiatives designed to reassure and facilitate knowledge about risk stratification and reduced screening of low-risk women.

#### Subtheme 2.3. Impact of mixed messaging and hearsay

When media coverage becomes less focused on changes to screening, women’s main sources of information will be their breast screening service, GP practice and other women in their communities. As HCPs considered how local services should communicate screening changes, they identified that messages for less frequent screening are not congruent with current advice (to attend routinely every 3 years). They explained that women would receive mixed messages from their service and would not be able to differentiate between them, potentially causing confusion and anxiety:

Some may feel anxious about it because it’s a change in what we’ve been hammering home for quite some time that you must have it every three years and then suddenly it changes so it might cause anxiety to some women. (GP: TI2)

Women would also hear about changes to the screening programme from those at low risk within their community, and women who have not been risk assessed could wrongly assume that they too do not need to attend screening as often. Some women could also assume they are at low risk when comparing themselves to those with low-risk results, raising concerns about women using the low-risk pathway as an excuse to attend screening less often or not at all:

… one thing I’m conscious of is that you’ve got ladies who don’t want to come and will find a new reason not to come, and if they think that some ladies are deemed low risk they may take the assumption that they’ve no family history so they must be low risk and they won’t come either. (Advanced Practitioner – Radiography: FG1)

HCPs, therefore, argued that introducing risk-stratified breast screening could create confusion for women if internal and external communications are mishandled.

### Theme 3 – Deliberating service implications and reconfiguration management

HCPs discussed the service-level implications of risk-stratified screening. Three major implementation areas were highlighted as requiring answers: (1) who qualifies for risk-stratified screening; (2) how will risk stratification fit within the existing service; and (3) what roles and responsibilities will HCPs have.

#### Subtheme 3.1. Prevalent vs incident round rollout

In the NHSBSP, a woman’s first mammogram is known as a ‘prevalent round’ screen. Subsequent screening mammograms are ‘incident round’ screens. When considering which cohort should be offered risk-stratified screening, HCPs pointed to the management of women’s expectations as a factor to consider. They were concerned that if low-risk ‘incident round’ women were to attend screening less frequently, existing ‘promises’ would be broken and could raise suspicions as to why the frequency at which they attend screening had been altered:

… it’s always easier to start with your new cohort going through and this is what’s going to happen. If you start changing things people are always more suspicious, I think halfway through. (GP: TI1)

With this in mind, HCPs suggested that prevalent round women would be the most appropriate group to be offered risk-stratified screening, and a low-risk pathway could be more acceptable to those entering the programme as these women may be less aware of the current screening frequency. However, HCPs also suggested that if risk-stratified breast screening was rolled out to prevalent round women only, the service would miss high-risk incident round women who could benefit from extra screening or preventive medications:

Because some of the people on the three-yearly may actually, if they’re risk assessed, have a higher risk and might need to be done more often. It’s not only about picking up people as low risk, is it? (GP: TI1)

Thoughts instead turned towards equity of access and if proven to be safe, all women in the programme should be offered risk-stratified screening.

#### Subtheme 3.2. Integrating a low-risk screening interval

HCPs discussed how low-risk extended screening intervals could fit into the present programme. A screening interval longer than 3 years would cause significant service disruption due to the loss of synchronization of these less frequent screening invitations with the 3-yearly rotation of the mobile screening units. To mitigate physical and logistical service impact, a 6-yearly screening interval was deemed more favourable, allowing low-risk women to be invited in alternate rounds:

But if we have another set of women that were saying that we’re going to invite every five years, logistically that’s going to be very difficult because where do we invite them to as far as where do they go for screening? (Breast Screening Office Manager: FG1)

You mean you’ll have to make it six years. They’ll be screened at the same place. (Consultant Radiologist: FG1)

So, they’d be screened at the same site, yeah. Because if you do it five years it doesn’t fall in with your three years and you have to come here [static screening site], and women don’t travel in this area. (Breast Screening Office Manager: FG1)

We don’t have the capacity to do it. (Advanced Practitioner – Radiography: FG1)

Nevertheless, HCPs explained that the interval length should ultimately be driven by scientific evidence and safety, rather than ease of integration into the current programme. To effectively implement multiple screening pathways, HCPs advised learning from other national programmes, for example, cervical screening where different pathways have been implemented successfully.

#### Subtheme 3.3. Delegation of responsibilities

Prior to implementation, a system should be established to manage women’s queries and concerns, although whose responsibility this should be was debated. It was suggested that radiography staff at the point of screening would not be appropriate due to time constraints within appointments. Alternatively, primary care staff were suggested to educate women on risk and manage queries about screening changes as GPs explained women would contact their surgery as their ‘first port of call’. However, it was identified that GPs receive large amounts of new information daily about changes across all NHS services. To avoid being lost among other notifications, it was suggested that practices should be supplied with information about risk-stratified breast screening when it becomes locally applicable:

I suppose the other thing in terms of breast screening is that it’s done in batches per practice so you are notified a few months’ ahead of time that your practice is going to be screened. That’s an opportunity to let the practice know about the changes at a time that’s relevant for them because that’s when their patients are likely to be notified and coming in, that’s an opportune time where it’s relevant. (GP: TI2)

HCPs in one focus group identified Breast Care Nurses as appropriately trained to have conversations with women, especially if women are required to make a choice about screening frequency. This option may not be viable because of workload considerations. To divert questions away from frontline staff, HCPs also suggested a helpline, but it was unclear who should manage this.

## Discussion

### Overview of findings

This study is the first, to our knowledge, to explore views regarding less frequent screening for low-risk women from the perspective of HCPs working in the NHSBSP and primary care. Overall, risk-stratified screening was viewed as a logical next step in the personalisation of breast cancer screening, with less frequent screening for low-risk women not opposed but discussed thoughtfully by the HCPs. Whether women at low risk could safely be screened less frequently was debated, raising doubts about the accuracy of risk estimation. HCPs described their discomfort in advocating less frequent screening for low-risk women due to the possibility of interval cancers. There was apprehension that low risk may be construed as no risk, resulting in reduced screening attendance and women paying less attention to symptoms. However, our previous work showed no reduction in screening attendance of women who were informed they were at low risk.^19^ Media coverage of risk stratification and less frequent screening was a particular concern, as well as the management of localized information and knowledge. HCPs debated to whom a risk-stratified screening service should be rolled out, with equity of access essential. Logistical issues were also discussed, including integrating a low-risk pathway into an established programme and establishing professional responsibilities for managing queries.

### Strengths and limitations

Strengths of this study include the recruitment of a varied sample of HCPs from three UK breast screening sites who are currently implementing risk-stratified screening as part of a feasibility study.^9^ This facilitated a holistic understanding of the possible impact of introducing risk-stratified screening with a low-risk pathway into the service, as staff were able to offer their views from the perspective of their differing roles and their experiences implementing risk stratification. The involvement of GPs also enabled an exploration of issues that could be encountered in primary care. This professional group represents a small proportion of the participant sample; however, it would be unlikely that such a risk-stratified programme would be implemented through primary care rather than the organized NHSBSP. A limitation of this research is that HCPs discussed the implications of a low-risk screening pathway hypothetically, without a tangible framework of how such a pathway could be implemented and it could be that participants held stronger opinions for or against low-risk extended screening intervals. Nevertheless, initial reactions to the proposal and identification of issues from those working in the NHSBSP and primary care are invaluable when considering how a low-risk pathway should be designed, communicated and implemented. However, should a low-risk pathway be implemented in future, it will be important to gain HCP views on the service design and implications for practice. Although HCPs were recruited from three distinct screening sites, the views expressed may not be representative of the wider breast screening population. For example, HCPs in this study currently run a risk-stratified service as part of a feasibility study^9^ and have offered their views regarding risk-stratified screening in previous focus groups.^25^ HCPs at less research-intensive units may therefore hold differing views about risk-stratified screening and a low-risk pathway. Similarly, HCPs in this sample work within the context of a publicly funded healthcare system; therefore, findings would likely be different in countries where breast screening is opportunistic.

### Relevance to existing research

A particular issue raised was whether women would interpret a low-risk estimate as them having no risk of developing breast cancer, especially if encouraged to attend screening less often. False reassurance from test results has been found in other disease areas. A cystic fibrosis study found that 50% of individuals who had screened negatively wrongly presumed themselves as having no risk of being a carrier when reflecting on their result 3 years later.^30^ However, a recent systematic review suggests no strong relationship between false reassurance and negative cancer screening test results.^31^ For instance, in two studies where a low-risk results were given for prostate and breast cancer, respectively, feelings of reassurance were not increased.^32,33^

HCP discomfort regarding conversations with women about risk estimation and preventive treatment options has been previously documented.^34–36^ In particular, HCPs have voiced unease towards prescribing preventive medications for breast cancer and thereby provoking anxiety.^32,33^ Driven by concerns about interval cancers and subsequent blame attribution, this study has shown that HCPs would also feel uneasy about facilitating decisions for less frequent screening if low-risk women are to decide how often they attend. This adds support to the opinion that although interval cancers will still occur in a minority of low-risk women, those unfortunate enough to develop breast cancer could feel let down by the service.^13^

HCPs questioned the accuracy of a 10-year breast cancer risk estimate, a valid issue considering that current breast cancer risk models undergo continual refinement to improve their discriminatory capacity.^37^ In this study, it was contested whether less frequent screening could be safely implemented due to risk factor instability and accuracy of self-report information. Concerns about providing accurate information have also been documented by women, causing them to question whether they can trust their risk estimate.^38^ HCPs pointed to an increased risk of breast cancer with age as one factor which could alter a low-risk estimate and although this is correct, older women are more likely to have a better prognosis than those who develop the disease at a younger age where cancers tend to be more aggressive.^39^ Nevertheless, whether reassessment of risk would be needed requires further exploration.

In accordance with the present literature,^13,21,38^ HCPs acknowledged that a low-risk screening pathway could encounter negative press and public opinion, if the service is perceived as ‘cost cutting’. Women in the United Kingdom generally hold positive views about breast screening^40^ and are used to a 3-yearly programme.^13^ An abrupt change to this message could cause scepticism. Nevertheless, a review of the UK screening programme^3^ which examined the evidence regarding the value of population-based screening could be used to highlight the message that a change to screening is needed for a better balance of harms and benefits, thus potentially reducing criticism.^13^ However, as mentioned here and elsewhere,^40^ women generally have a limited understanding of the harms of breast screening and without direct attention given to the explanation of harms women could continue to view less frequent screening negatively.

Risk-stratified screening could require additional contact time with the service.^13^ This was appreciated in the present study, with HCPs acknowledging that women would require designated personnel to answer their questions and deemed especially important if low-risk women are required to choose their screening interval. To whom this responsibility should fall remains to be ascertained although preferences for a risk expert have been suggested by women.^41^ GPs have also been cited by some women as professionals to provide risk feedback information.^41^ However, for women in the UK, GP knowledge and their ability to provide risk feedback has been contested.^42^

### Implications for practice and future research

Before implementing a risk-stratified screening service with a low-risk pathway into research studies and clinical practice, there are a number of key issues identified by HCPs which need to be addressed where further research may be required.

It will be necessary to provide HCPs with the appropriate evidence around accuracy of risk-stratified screening to satisfy the need to demonstrate the safety of introducing reduced screening intervals for those at lower risk. This should include research data indicating that low-risk women are not at higher risk of high-grade interval cancers. In addition, HCPs will be a useful stakeholder group to provide insight into developing public communication strategies that will engage with those who are invited to their service.

To which population of screening aged women risk-stratified screening should be rolled out to will need to be determined, whether this be prevalent round only, incident round or all. When considering implementation, it will be important to remain cognisant of the implications for all risk groups, not just those at low risk. Further research is required to explore the optimum strategy for implementing risk-stratified screening to enable the maximum number of women to benefit from this service, while managing the expectations of those who may feel disadvantaged by less frequent screening. Additional research may also wish to explore stakeholder views regarding a later start to screening for women at low risk as a possible strategy.

Whether women would need a further risk reassessment within or at the 10-year period requires exploration. It has been suggested that monitoring modifiable risk factors would allow estimates to remain up to date.^34^ Further research should explore the relationship between risk estimates and changes to risk factors to establish whether a reassessment pathway would be required and, if required, how it should be implemented.

Piloting risk stratification with low-risk screening will be essential to establish gaps in HCP training and knowledge, as well as to highlight questions women may ask. If women at low risk are expected to make a choice about their screening, Patient Decision Support Tools^36^ could help HCPs attain a level of detachment from women’s decision making, potentially reducing the feeling of being personally responsible for the decision made. However, whether low-risk women should be provided with a choice of screening frequency has yet to be examined. Should a choice be given, future research should explore the extent of an HCP’s role in the decision making process, as well as establishing the support needs for managing women’s queries.

To mitigate adverse opinions of the breast screening service, clear communication will be needed between the service and the press to provide the public with transparent information about risk stratification. To increase public knowledge, the service should consider working with the media to publicize the harms and benefits of breast screening, as well as the rationale for low-risk screening. Public education campaigns could prove effective here.

Finally, the literature remains inconclusive as to whether women at low risk would view themselves as having no risk of developing breast cancer and has yet to be explored in depth. Future research should aim to examine women’s perceptions of being at low risk of breast cancer and identify implications for future screening attendance and personal surveillance of symptoms. In addition, exploring low-risk women’s views regarding less frequent screening would provide insight into service user acceptability, which could inform service development.

## Conclusion

Although perceived as the logical next step, HCPs did have key concerns about less frequent screening for women at low risk which would need to be considered before implementation. The accuracy of risk estimates needs to be ascertained to instil trust in HCPs and women. Whether women at low risk should be provided with a choice of screening frequency requires further study. Defining communication responsibilities and supporting HCPs to feel confident in having conversations with low-risk women will be essential. When considering eligibility criteria for risk-stratified breast screening, implementation should consider options that benefit the maximum number of women across all risk groups, despite whether a low-risk pathway would draw criticism. Communication from the service will need to clearly emphasize to women that low risk does not mean no risk, and remaining vigilant of symptoms and attending subsequent screens continues to be important. Navigating press and public scrutiny will need to be explicitly managed through communication and public education campaigns to enhance knowledge. Finally, how a risk-stratified breast screening service with a low-risk pathway should be integrated into an existing programme requires further exploration with explicit focus on logistical issues and service capabilities.

## Supplemental Material

sj-pdf-1-whe-10.1177_17455065211009746 – Supplemental material for Introducing a low-risk breast screening pathway into the NHS Breast Screening Programme: Views from healthcare professionals who are delivering risk-stratified screening Click here for additional data file. Supplemental material, sj-pdf-1-whe-10.1177_17455065211009746 for Introducing a low-risk breast screening pathway into the NHS Breast Screening Programme: Views from healthcare professionals who are delivering risk-stratified screening by Victoria G Woof, Lorna McWilliams, Louise S Donnelly, Anthony Howell, D Gareth Evans, Anthony J Maxwell and David P French in Women’s Health
