# Supplementary material for: Introducing a low-risk breast screening pathway into the NHS Breast Screening Programme: Views from healthcare professionals who are delivering risk-stratified screening
Source: Womens Health (Lond). 2021 Apr 20;17:17455065211009746. doi: 10.1177/17455065211009746 (PMC8060757; doi:10.1177/17455065211009746)
Supplement: sj-pdf-1-whe-10.1177_17455065211009746 – Supplemental material for Introducing a low-risk breast screening pathway into the NHS Breast Screening Programme: Views from healthcare professionals who are delivering risk-stratified screening [file sj-pdf-1-whe-10.1177_17455065211009746.pdf]

**Supplemental Material Topic Guide**

# Healthcare professional's views on increasing the screening interval for women at low risk of developing breast cancer.

## Discussion points

These discussion points are related to the aims of the interviews:

- How would you like to see this implemented if an increased screening interval for low risk women was introduced?
- What is the feasibility of introducing an increased screening interval beyond 3 years for low risk women into the NHSBSP?
- What factors should inform an increased screening interval for low risk women? Cost of screening? Number of missed cancers? Number of overdiagnosis? Risk Threshold.
- What risk threshold should be used to recommend an increased screening interval?
- As a HCP who works in the screening service what threshold of risk would you be comfortable with discussing with a woman who may be eligible for an increased screening interval? (Prompt: For example you could have women at two extremes of the category, i.e. at 0.5% and 1.5%.) Context – if a women were to ask what you think etc.
- What would be an acceptable length between screening intervals?
    - When should it be introduced (prompt: age of 1st screen)
    - When should risk be re-assessed?
- It is hoped that stratified screening for low risk women will cost less and screening for high risk women will cost more. How do you feel about that? How comfortable are you with this? What financial implications do you foresee with the proposal of risk stratified screening?
- In thinking about implementation, what issues could you encounter? (prompt: Complaints; Queries; Who is the contact for questions? How could these be mitigated?
- What information will women need to make a decision about their screening and in what form should they receive this information?
- As HCPs working in the NHSBSP what advice would you be comfortable in giving women? Who should be giving the advice?
- What should women know about why reducing the frequency at which they attend for screening is being recommended, i.e. because of the cost of high risk screening? Harms of screening?
- How should the information be communicated?
    - Via letter?
    - Face to face?
- How would women opt for this change in their screening?
    - Opt in or opt out option?
- What are benefits of having an increased screening interval for low risk women?
- What are the potential issues associated with an increased screening interval for low risk women? How could these be mitigated?
- What psychological impact could reducing the frequency at which women attend for screening have on low risk women? With these factors in mind how comfortable would you feel in discussing this change with women?
- What is the best way to facilitate informed choice? (prompt: own decision; HCP advice; PHE advice; both)
- Do you foresee this change biasing choice? Prompt – Effecting uptake?
- How can different groups of women be supported? SES, BAMER?
- What do you think is the most important consideration for implementing risk stratified screening for low risk women?
